# The neuro-immune axis in preeclampsia: from the maternal-fetal interface to systemic dysregulation

**DOI:** 10.3389/fimmu.2026.1761197

**Published:** 2026-04-17

**Authors:** Jingting Liu, Yue Zhao, Chong Zhang, Jianying Pei, Yan Li

**Affiliations:** 1 Clinical Laboratory Center, Gansu Provincial Maternity and Child-care Hospital, Lanzhou, China; 2 Operation Management Department, Gansu Provincial Maternity and Child-care Hospital, Lanzhou, China; 3 Department of Biochemistry and Molecular Biology, Medical College of Northwest Minzu University, Lanzhou, China

**Keywords:** immune system, maternal-fetal interface, nervous system, neuro-immune axis, preeclampsia

## Abstract

Preeclampsia (PE) is a complex hypertensive disorder of pregnancy characterized by new-onset maternal hypertension and multi-organ dysfunction. Although placental maladaptation and immune activation are well-established features of PE, growing evidence indicates that dysregulated neuro–immune–vascular integration critically contributes to disease initiation, progression, and long-term sequelae. Normal pregnancy requires coordinated immune and neural adaptations, particularly at the maternal–fetal interface, to support successful placentation. While placental pathology, angiogenic imbalance, and immune activation establish the systemic environment of PE, some neurological phenotypes (such as eclampsia and acute cerebral autoregulatory failure) are difficult to explain without involvement of central autonomic and sensory integration circuits that mediate the translation of peripheral inflammatory and vasoactive signals into neurovascular responses. Dysfunction of cerebral autoregulation has been proposed as a key mechanism underlying acute neurological complications, independent of classic placental factors. In PE, this finely tuned communication becomes spatially and functionally disrupted, triggering cascades of inflammatory and vascular pathology. Emerging studies suggest that neural signals, including autonomic activity and neuropeptide signaling, may modulate local immune phenotypes and vascular responses, thereby sustaining feed-forward cycles of inflammation and endothelial dysfunction. Altered neural inputs to peripheral immune organs may further bias myelopoiesis and amplify systemic inflammatory burden. At the central nervous system level, persistent neuroinflammation and blood–brain barrier disruption may potentiate systemic inflammatory signals, contributing to acute neurological manifestations and increased long-term cerebrovascular risk in women with prior PE. This review synthesizes evidence from human studies and experimental models to delineate neuroimmune mechanisms implicated in PE, identifies critical gaps in current knowledge, and highlights emerging concepts such as neuroimmune memory and neuro–metabolic crosstalk. We further discuss translational opportunities, including biomarker discovery, neuro-modulatory interventions, and advanced approaches such as single-cell and spatial omics. By integrating classical immunovascular paradigms with emerging neuroimmune insights, we propose a more comprehensive framework for understanding PE pathogenesis and for developing novel diagnostic and therapeutic strategies.

## 1 Introduction

Preeclampsia (PE) is a complex, multisystem pregnancy disorder, affecting 5–8% of pregnancies globally, remains a leading cause of maternal and perinatal morbidity and mortality (1, 2). Despite extensive research over the past decades, its precise etiology and pathophysiology are still not fully elucidated, hindering the development of effective preventive and therapeutic strategies beyond symptomatic management and delivery (2, 3). The initial conceptualization by James Young in 1914, linking placental infarctions to maternal toxin release, and later by Roberts and Taylor in the 1980s, identifying PE as an endothelial disease, laid foundational groundwork (2). However, the seminal work linking elevated soluble fms-like tyrosine kinase 1 (sFlt1) levels to PE (2), has unveiled a far more nuanced pathophysiology, highlighting the placenta as the primary instigator and the maternal systemic response as the ultimate determinant of clinical presentation and severity (4, 5). Historically defined by hypertension and proteinuria, the understanding of PE has evolved dramatically, transcending a purely vascular disorder to encompass a complex systemic syndrome involving intricate interactions between the immune, nervous, and vascular systems (2, 6, 7). Previous studies showed that, the dominant pathophysiologic model places the placenta at the origin of disease: defective trophoblast invasion and inadequate spiral artery remodeling generate placental hypoxia and oxidative stress (8), driving release of anti-angiogenic factors (sFlt-1 (9), soluble endoglin (10)), inflammatory mediators (11), and microparticles into the maternal circulation; these lead to systemic endothelial dysfunction and hypertension (4). This placental–endothelial axis explains many clinical features of PE but leaves open how fast, system-wide maternal responses (abrupt pulmonary edema, seizures, rapid blood pressure escalation) occur and why some pregnancies progress to severe maternal multi-organ disease (5, 12). While the classical model successfully identifies placental ischemia and the subsequent release of anti-angiogenic factors (e.g., sFlt−1, soluble endoglin) as initiators of endothelial injury, it remains insufficient in explaining the kinetic amplification of systemic inflammation and the postpartum persistence of autonomic dysregulation.

Neuro-immunology, centered on the bidirectional crosstalk between the nervous and immune systems (13), offers mechanistic insights that complement the limitations of the classical placental-angiogenic model. This new way of thinking shows we must study how different body systems work together. In this context, the neuro-immune axis is a key player (14, 15). A neuro-immunological framework offers complementary insight into autonomic and neural–immune interactions that may link placental signals with systemic phenotypes. It normally helps keep the body in balance during pregnancy, but when it functions poorly, it becomes an important factor in PE. The nervous and immune systems form an integrated network, constantly communicating to modulate immune cell characteristics and maintain tissue homeostasis (16). In pregnancy, this neuro-immune interplay is vital at the maternal-fetal interface for establishing immune tolerance and ensuring adequate placentation (17–19). Dysregulation within this axis is increasingly implicated in PE, contributing to systemic inflammation, oxidative stress, and endothelial dysfunction (20). The initial placental pathology, stemming from inadequate trophoblast invasion, triggers the release of anti-angiogenic factors and pro-inflammatory mediators, which interact with the nervous system to create a vicious cycle exacerbating endothelial damage and organ dysfunction (19, 20). The initial placental pathology, often stemming from inadequate trophoblast invasion and subsequent placental ischemia, triggers the release of anti-angiogenic factors (5) and pro-inflammatory mediators into the maternal circulation (2, 21). This systemic inflammatory milieu, in turn, interacts with the nervous system, creating a vicious cycle that exacerbates endothelial damage and contributes to the widespread organ dysfunction observed in PE (20). Understanding the precise mechanisms of this neuro-immune dysregulation, from its spatial origins at the maternal-fetal interface to its functional consequences on immune cell behavior and systemic impact, is paramount for developing effective diagnostic, preventive, and therapeutic strategies.

This review aims to comprehensively explore the neuro-immune axis in PE, dissecting its physiological basis during normal pregnancy, elucidating the spatial and functional dysregulations, and to explore new research and treatments. By integrating recent findings, we offer a comprehensive understanding of PE as a neuro-immune-vascular disorder to guide future research and improve patient care.

## 2 The neuro-immune regulation in healthy pregnancy

Pregnancy represents a unique physiological state requiring profound adaptations across multiple maternal systems, including the neuro-immune axis, to ensure successful fetal development and maternal health (18). The maternal-fetal interface is a site of intense immunological activity, where the maternal immune system must tolerate the semi-allogeneic fetus while maintaining protection against pathogens (18, 22). This intricate balance is partly regulated by neuro-immune communication.

### 2.1 Adaptive reprogramming in normal pregnancy

The maternal physiological landscape undergoes significant adaptive reprogramming, balancing immune tolerance to the fetus with protective immunity against pathogens (23). The nervous system plays a pivotal role in modulating these immune adaptations, influencing immune cell development, distribution, and effector functions (14, 16). For instance, neural signals can directly impact hematopoietic processes in the bone marrow, thereby shaping the immune cell repertoire available for pregnancy-specific tasks (16). Furthermore, the autonomic nervous system also helps control inflammation. Specifically, the vagus nerve can reduce inflammation in organs like the spleen. It does this by calming immune cells and controlling cytokine release. This process is important for keeping the immune system stable during a healthy pregnancy (24). This adaptive reprogramming is not static but dynamically changes throughout gestation, reflecting the evolving needs of the maternal-fetal unit. The successful establishment of pregnancy relies on a finely tuned neuro-immune communication that ensures the uterus is receptive to implantation, the placenta develops correctly, and the maternal system can sustain the pregnancy without detrimental inflammatory responses.

### 2.2 Immune system

During normal pregnancy, the maternal immune system undergoes a complex shift towards a Th2-dominant cytokine profile (18). Key immune cells at the maternal-fetal interface include decidual natural killer (dNK) cells (25), macrophages (Mφ) (26), and regulatory T cells (Tregs) (27). This involves a nuanced balance, often characterized by a shift towards a Th2-dominant cytokine profile, which is generally considered more tolerogenic, while still retaining sufficient Th1 responses for pathogen defense (18, 26, 27). For instance, dNK cells are abundant in the decidua and play a critical role in uterine spiral artery remodeling and trophoblast invasion, processes essential for adequate placental perfusion (25, 28). Their function is tightly regulated by interactions with trophoblasts and other decidual immune cells, often involving specific receptor-ligand interactions like KIR-HLA-C (29, 30). Mφ, particularly Hofbauer cells (HBCs) in the placental villi, typically adopt an M2-like phenotype in healthy pregnancy (26, 31), contributing to tissue remodeling, angiogenesis, and immune suppression, rather than pro-inflammatory M1 activation (26, 31, 32). Tregs are also significantly expanded and activated during normal pregnancy, crucial for suppressing maternal immune responses against fetal antigens and maintaining immune tolerance (27). The functions of these immune cells are guided by neural signals and neuropeptides (33). This control is vital, as it directly influences the cells’ development, their level of activation, and the cytokines they produce (34). Through this process, the nervous system helps shape a supportive immune environment for pregnancy.

### 2.3 Nervous system

The nervous system exerts profound control over immune responses, a concept increasingly appreciated in pregnancy. The autonomic nervous system, through its sympathetic and parasympathetic branches, is a primary mediator of neuro-immune communication (14). Sympathetic nervous system (SNS) fibers innervate lymphoid organs, releasing neurotransmitters like norepinephrine that modulate immune cell activity, proliferation, and trafficking, influencing macrophage polarization and cytokine secretion (16, 35). For instance, norepinephrine can influence macrophage polarization and cytokine secretion, contributing to the anti-inflammatory environment required for pregnancy. Conversely, the parasympathetic nervous system (PSNS), via the vagus nerve, activates the cholinergic anti-inflammatory pathway, releasing acetylcholine to dampen excessive immune responses (23, 36). This neural regulation extends to the central nervous system, which integrates systemic signals and influences immune responses through neuroendocrine pathways and direct neural circuits (36). During normal pregnancy, this neuro-immune axis is finely tuned to support unique immunological demands, ensuring appropriate immune modulation to prevent fetal rejection while maintaining maternal health. Neuropeptides further highlight this intricate interplay at the maternal-fetal interface (33, 37). The integrity of these neuro-immune circuits is essential for the adaptive reprogramming of the maternal system, and any disruption can have significant consequences for pregnancy outcome.

## 3 Spatial dysregulation of neuro-immune signaling in preeclampsia

PE is traditionally regarded as a pregnancy-specific syndrome that resolves following placental delivery. However, more epidemiological and clinical evidence indicated that PE confers substantial long-term risks to cerebrovascular and cognitive health later in life. A large cohort study demonstrated that women with a history of PE have a significantly increased lifetime risk of stroke compared with women without hypertensive disorders of pregnancy (38). In addition, observational cohort data showed that former PE patients exhibit subtle but clinically meaningful impairments in psychomotor speed and executive function many years postpartum (39). PE is fundamentally characterized by a profound spatial dysregulation of the neuro-immune axis, originating at the maternal-fetal interface and propagating throughout the maternal system (4). This disruption alters the delicate balance required for healthy pregnancy, leading to systemic inflammation, endothelial dysfunction, and multi-organ involvement (20) (**Table 1**). Understanding these aberrant interactions is crucial for identifying PE pathogenesis.

**Table 1:** Comparison of mechanistic models in preeclampsia pathogenesis.

Model	Key features explained	Limitations/mechanistic gaps
Placental Angiogenic Imbalance	sFlt-1/PlGF imbalance disrupts VEGF signaling; Endothelial dysfunction and systemic vascular resistance	Cannot fully explain neuro-immune feedback; Cannot mechanistically link placental signals to systemic neural regulation
Maternal Immune Dysregulation	Aberrant maternal immune responses (Th1/Th17, Treg); Systemic inflammation and endothelial damage	Focuses on immune dysregulation without incorporating autonomic neural modulation; Cannot specify how neural circuits shape immune phenotypes
Oxidative Stress & Redox Imbalance	ROS accumulation in placenta damages tissues; Enhances anti-angiogenic factor release	Primarily biochemical, lacks pathways explaining systemic neural control or CNS contributions
Neuroimmunological Integration	Includes autonomic (sympathetic/vagal) regulation of immune cells; Recognizes neural involvement in systemic inflammation and blood pressure regulation; Potential neurogenic influences on immune trafficking and vascular tone	Direct causal evidence in PE models still emerging; Needs future experiments to establish neural causality

### 3.1 Maternal-fetal interface: the primary hub for neuro-immune communication

The maternal-fetal interface is the initial site where the neuro-immune axis goes awry in PE, setting the stage for systemic pathology (18, 40). While clinical evidence confirms that in normal healthy pregnancy, this interface is a finely tuned environment for immune tolerance and spiral artery remodeling (17, 19). However, preclinical models suggest that a disruption in this intricate crosstalk precipitates inadequate trophoblast invasion and placental hypoxia (2, 41). This initial placental dysfunction drives the maternal syndrome by releasing anti-angiogenic factors like sFlt-1 (9) and pro-inflammatory mediators into the maternal circulation (2, 21, 42). Correlative insights from single-cell transcriptomics and spatial multi-omics have provided high-resolution signatures of these alterations. Human decidual studies reveal cell-type-specific gene expression alterations, including dysregulated angiogenic factors and inflammatory signatures (30, 43). For example, single-cell RNA sequencing (scRNA-seq) identified IGFBP1^+^SPP1^+^ extravillous trophoblasts (EVT) with immune cellular dysfunction and reduced HLA-F expression in PE decidua (30, 44). PE also shows an excess of EVTs and fewer mesenchymal and HBCs, driving bulk gene expression differences like FLT1, LEP, and ENG overexpression (45), they primarily provide descriptive associations rather than direct functional evidence of neural-driven function. However, direct *in vivo* evidence that neural or neuropeptidergic signaling regulates dNK cell or decidual macrophage function in human PE is lacking. Although neuro-immune interactions have been mechanistically demonstrated in non-pregnant systems and animal models, extension of these findings to human PE remains largely inferential. Future mechanistic studies are needed to determine whether neuro-immune pathways contribute additively or interactively with cytokine networks at the maternal–fetal interface in PE.

Immune cells at this interface exhibit significant dysregulation. dNK cells show abnormalities in quantity, phenotype, and function, contributing to insufficient activation and impaired vascular transformation (25). Mφ, including HBCs and decidual Mφ (dMφ), display aberrant polarization patterns. While HBCs in normal pregnancy are M2-like, in PE, they can exhibit pro-inflammatory M2 phenotypes, contributing to altered tissue homeostasis (26, 46). In animal models, a specific CD11c high decidual macrophage subset, activated by galectin-9 from trophoblasts, has been shown to inhibit spiral artery remodeling in PE (47). T cells also show altered cytokine production, informing immunotherapeutic strategies (48). Even the maternal gut microbiota has been implicated, with dysbiosis influencing immune activation at the maternal-fetal interface, suggesting a neuro-immune-metabolic link (49). PE is characterized by a dysregulated immune milieu marked by Th1 dominance, increased placental release of pro-inflammatory cytokines, and aberrant activation of macrophages and dNK cells, collectively sustaining a pro-inflammatory environment that amplifies immune activation (50). Elevated inflammatory cytokines, particularly tumor necrosis factor-α (TNF-α) and interleukin-6 (IL-6), contribute to widespread maternal endothelial dysfunction, which is thought to underlie the development of hypertension (51).

Another core issue is overactive sympathetic nerves at the maternal-fetal interface. This local problem connects to body-wide sympathetic nervous system overactivity, which together cause key symptoms like hypertension and vascular dysfunction (52). This systemic overactivity likely translates to local effects, where neurotransmitters from sympathetic nerve endings modulate resident immune cells and vascular smooth muscle cells. Norepinephrine can influence macrophage polarization, cytokine production, and immune cell migration, potentially exacerbating the pro-inflammatory environment and impairing trophoblast invasion (14, 16). Increased sympathetic tone could shift macrophage polarization towards pro-inflammatory M1 or specific M2 subtypes (24, 26), contributing to impaired spiral artery remodeling. Furthermore, sympathetic signals can directly impact vascular tone and remodeling. Overactivity could lead to vasoconstriction of the spiral arteries, further compromising placental perfusion and exacerbating the hypoxia-ischemia (41). The interplay between sympathetic nerve signals and local immune cells at the maternal-fetal interface could create a self-perpetuating cycle: placental ischemia triggers inflammation, which in turn activates sympathetic pathways, further impairing placental function and amplifying systemic inflammatory responses. The complex interactions between neural signals, immune cells, and vascular remodeling at this interface highlight its central role in the pathogenesis of PE, making it a prime target for future research and intervention.

### 3.2 Peripheral immune organs and circulation: remote controllers of neural signaling

The dysregulation starts at the maternal-fetal interface in PE, then, quickly spreads to peripheral immune organs and into the blood. In turn, these sites become hubs that receive and amplify the faulty neural signals (4, 20). This process ultimately worsens the disease. The nervous system maintains extensive connections with peripheral immune organs, modulating immune cell development, distribution, and function (23), and these connections become pathologically altered in PE. The systemic inflammatory state, characterized by a “cytokine storm” and endothelial dysfunction (53), is not merely a consequence of placental factors but is actively shaped by neuro-immune interactions in these peripheral sites (20).

#### 3.2.1 Bone marrow

The bone marrow (BM), the primary site for hematopoiesis, is profoundly influenced by neural signals (16, 54). Sympathetic nerve fibers innervate the bone marrow and locally release neurotransmitters such as norepinephrine, which regulate hematopoietic stem cell (HSC) mobilization and niche function (55). While direct evidence of altered sympathetic innervation specifically in the BM of human PE patients is currently lacking, the role of the BM as a “remote controller” is supported by preclinical models and indirect clinical markers. In spontaneously hypertensive rats, increased BM norepinephrine and altered sympathetic activity correlate with increased inflammatory precursors and reduced progenitor cells, supporting a link between SNS activity and BM immune output (56).

In PE, systemic inflammation and stress, which often associated with heightened sympathetic activity, are likely to influence BM function and immune cell development. Chronic inflammatory exposure during PE may alter the behavior of BM stem and progenitor cells, potentially leading to dysregulated hematopoiesis and innate immune output. Sympathetic signaling via norepinephrine has been shown in other inflammatory contexts to promote myeloid expansion, increasing the production of myeloid-derived immune cells such as neutrophils and monocytes, cell populations that are consistently elevated and activated in PE (57). A bias toward a pro-inflammatory myeloid phenotype within the BM could therefore contribute to systemic inflammation and provide a source of activated immune cells capable of infiltrating target organs and aggravating endothelial dysfunction (58). Collectively, these observations support the hypothesis that neuro–immune crosstalk regulating hematopoiesis may be perturbed in PE, possibly involving epigenetic reprogramming or senescence-related pathways. In this framework, the BM may function as a remote immunoregulatory organ that translates systemic neuroinflammatory signals into altered immune cell output, rather than as a site of primary pathology.

#### 3.2.2 Spleen

The spleen is a major secondary lymphoid organ that plays a crucial role in filtering blood, mounting immune responses, and serving as a reservoir for immune cells (59). In systemic inflammatory states, neural circuits influencing the spleen have been shown to modulate immune responses through a combination of sympathetic and parasympathetic mechanisms (24). This neuro-immune pathway has been described in endotoxemia and other inflammatory models, where electrical or chemical stimulation of autonomic circuits results in local norepinephrine (NE) release from the splenic nerve and subsequent modulation of immune cell cytokine output. Voltammetric recordings in rodents demonstrated that splenic NE release is responsive to autonomic neurostimulation and associated with suppressed pro-inflammatory responses, consistent with an anti-inflammatory reflex mediated through neural control of splenic immunity (60). Moreover, mechanistic study involving splenic nerve discharge showed that sympathetic activation via hypertensive challenges can prime peripheral immune responses and promote T cell activation and egress from the spleen in animal models (61). Notably, the vagus nerve did not directly innervate the spleen (62). Instead, central cholinergic efferented modulate splenic immune function indirectly through multisynaptic pathways involving celiac ganglia and splenic sympathetic fibers, which release NE to influence splenocyte behavior (63).

While these mechanistic insights provide a biologically plausible framework for neuro-immune modulation of splenic responses, direct evidence of altered splenic neural activity, innervation density, or neurotransmitter signaling in human PE is currently unavailable. In the context of PE, systemic inflammation and sympathetic overactivity are well described, and changes in circulating cytokines and immune cell mobilization are consistent with altered immune organ output (64). However, whether these phenomena reflect primary neural dysregulation of the spleen versus secondary responses to systemic factors remained to be determined. Building on established neuro-immune circuits in other inflammatory diseases, splenic neural pathways represent testable and translatable mechanisms in experimental preeclampsia. Targeted neural or pharmacological modulation of splenic neuro-immune signaling in PE models could be used to evaluate downstream inflammatory responses and disease-relevant outcomes. These approaches extend current concepts of neural regulation of inflammation into a translational framework and generate empirically testable hypotheses for future investigation.

### 3.3 Central nervous system: amplifiers and effectors of systemic inflammation

While PE is often initiated by placental dysfunction, its systemic inflammatory and vascular consequences profoundly impact the central nervous system (CNS) (4). Initially, it was believed that the brain was immune-privileged and isolated from the rest of the body (65). However, it is now established that the brain actively communicates with the peripheral immune system (66), and this crosstalk plays a key role in health (66, 67). In PE, this communication is critically disrupted, which drives the development of neurological symptoms and complications.

#### 3.3.1 Central nervous system inflammation

The systemic inflammation characteristic of PE, driven by placental factors and peripheral immune dysregulation, directly impacts the CNS, leading to neuroinflammation. Pro-inflammatory cytokines, anti-angiogenic factors, and other circulating mediators can cross the blood-brain barrier (BBB), which itself can become compromised in PE due to endothelial dysfunction (66). Once in the brain, these factors activate resident immune cells, primarily microglia and astrocytes, initiating a neuroinflammatory cascade. In PE, systemic endothelial dysfunction often extends to the cerebral vasculature, compromising the integrity of the BBB. This secondary leakage allows the passive influx of circulating pro-inflammatory cytokines and anti-angiogenic factors into the cerebral parenchyma. A study showed that microglial activation in the hypothalamus has been linked to metabolic dysregulation, including insulin resistance, and similar pathways may be implicated in PE-related CNS changes (68). Cerebral endothelial cells (CEC), which form the BBB, express innate immune receptors like toll-like receptors (TLRs) and inflammasomes, and their activation can compromise BBB integrity and promote neuro-immune interactions, modulating both systemic and neuroinflammation (66). An observational study in women with eclampsia reported elevated levels of claudin-5 (CLDN5) and matrix metalloproteinase-9 in cerebrospinal fluid, indicating disruption of cerebrovascular tight junctions and BBB injury (69). These patients also exhibited acute neuroinflammation, reflected by increased concentrations of inflammatory recruitment, cytotoxic, and immune-modulating markers in cerebrospinal fluid (69). Importantly, cerebrospinal fluid inflammatory marker levels did not correlate with plasma levels, suggesting that the neuroinflammatory response originates within the brain rather than resulting from passive leakage of systemic inflammatory mediators across the BBB.

This neuro-inflammation can manifest as cerebral edema, vasogenic edema (70), and white matter changes (71), which are often observed in neuroimaging studies of women with PE and eclampsia. The activation of inflammatory pathways within the brain can lead to neuronal dysfunction and damage, contributing to the neurological symptoms of the disease. In turn the brain can amplify systemic inflammation through neuroendocrine pathways, such as the hypothalamic-pituitary-adrenal (HPA) axis, and by modulating autonomic nervous system outflow, creating a vicious cycle of inflammation and CNS dysfunction (72). The concept of “gateway reflexes” describes how immune cells can bypass the BBB and infiltrate the CNS, causing neuroinflammation in various autoimmune diseases (73), and similar mechanisms might be at play in PE, allowing peripheral immune cells to contribute to brain pathology.

#### 3.3.2 Pathological behavior: from passive response to active amplification

The neurological manifestations of PE represent a transition from a passive CNS response to active inflammatory amplification. Traditionally, CNS involvement has been viewed as a passive responder to systemic hypertension and circulating anti-angiogenic factors. Symptoms such as severe headaches, visual disturbances, and hyperreflexia are seen as direct consequences of impaired cerebral autoregulation and increased BBB permeability (74), allowing the influx of systemic cytokines and proteins into the cerebral parenchyma (72). A study demonstrated that small extracellular vesicles (sEVs) derived from preeclampsia plasma or hypoxic placentas directly disrupt the BBB by reducing the expression of the tight junction protein CLDN5. These vesicles reach brain endothelial cells *in vivo*, induce regional BBB leakage (particularly in posterior brain areas) and were associated with neurological impairment. Although sEVs from preeclampsia were enriched in VEGF, the downregulation of CLDN5 occurred independently of VEGFR2/KDR activation, suggesting alternative vesicle-mediated regulatory mechanisms (75).

However, emerging evidence suggests that the CNS subsequently transitions into an active amplifier of the disease. We define this “active” role based on three key criteria:

First, neuroinflammatory activation (evidenced by microglial activation markers, altered cerebrospinal fluid cytokine profiles, and BBB dysfunction) has been documented in severe preeclampsia and eclampsia and may occur disproportionately to the degree of peripheral inflammation. CNS dysfunction in preeclampsia is not confined to the acute gestational period; women with a history of preeclampsia exhibit a significantly elevated long-term risk of stroke, cognitive impairment, and neuropsychiatric disorders (76), suggesting that the neuroinflammatory changes initiated during pregnancy can have lasting consequences. Second, the concept of neuro-immune memory provides a framework to reconcile these findings. Inflammatory and vascular insults during PE prime CNS-resident immune cells or neurovascular units, resulting in exaggerated or maladaptive responses to subsequent inflammatory, metabolic, or vascular challenges later in life (77). Such priming fulfills a key criterion of an active amplifier: durable reprogramming of CNS immune function that shapes future systemic or neurological outcomes, rather than a transient, reactive inflammatory state. Thirdly, Additional support for active CNS participation comes from emerging evidence implicating gut–brain–immune interactions in anxiety, depression, and cognitive changes (78). Systemic inflammation and stress may perturb brain endothelial microRNA profiles and neuro-immune signaling pathways, potentially reinforcing central inflammatory circuits even after pregnancy ends. While direct evidence remains limited, these observations suggest that CNS involvement in PE extends beyond passive exposure to circulating mediators.

Consequently, the CNS acts not just as an effector manifesting neurological symptoms, but as a “biological regulator” that amplifies and perpetuates the inflammatory cycle of PE. This distinction is vital for developing targeted neuro-protective therapies that address the brain’s active pathological role rather than just managing systemic blood pressure.

#### 3.3.3 Neural mediation of neurological phenotypes in PE

Although placental ischemia, immune activation, and endothelial dysfunction are central to the pathogenesis of PE, these mechanisms did not fully explain abrupt neurological manifestations such as seizures and acute failure of cerebral autoregulation. Cerebral autoregulation refers to the brain’s ability to maintain stable perfusion when systemic blood pressure changes. In eclampsia, this regulatory capacity is impaired, resulting in cerebral hypoperfusion, compromise of the BBB, neuroinflammation, and vasogenic edema (79).

Eclampsia is the most severe neurological complication of PE, characterized by the occurrence of generalized tonic-clonic seizures in a woman with PE without other neurological conditions (80). These seizures are a direct manifestation of profound CNS dysregulation and neuroinflammation (4). Comparative clinical studies of eclampsia versus PE without seizures showed evidence of blood–brain barrier disruption and elevated neuroinflammatory markers, suggesting that central neural regulation may be implicated in seizure pathogenesis (69). In PE and particularly in eclampsia, cerebral blood flow autoregulation is significantly impaired compared with preeclampsia without severe features or normotensive pregnancy, indicating a neural–vascular dysregulation that likely goes beyond placental ischemia alone (81, 82). While the exact mechanisms are not fully understood, they are believed to involve a combination of cerebral vasospasm (70), endothelial dysfunction (83), breakdown of the blood-brain barrier (72), and subsequent cerebral edema, leading to neuronal hyperexcitability. The systemic inflammatory milieu, with its elevated levels of pro-inflammatory cytokines and anti-angiogenic factors, contributes significantly to this cerebral pathology. These mediators can directly affect neuronal excitability and synaptic function, lowering the seizure threshold (84). The rapid onset and severity of eclamptic seizures underscore the critical role of the CNS as an effector organ in PE, where systemic dysregulation culminates in acute and life-threatening neurological events. In PE, autonomic dysfunction characterized by altered sympathetic and parasympathetic balance has been frequently reported, suggesting that central autonomic and sensory pathways modulate systemic cardiovascular and neurovascular responses associated with acute neurological phenotypes (79).

Hence, we propose that neural regulation functions as a phenotype-dependent mediator that is necessary for the expression of specific acute neurovascular outcomes, rather than a universal driver of all PE manifestations. This conceptual framework synthesizes current human and preclinical evidence, stratifies mechanistic hypotheses by support level, and identifies tailored, model-dependent predictions to guide future translational and clinical research.

## 4 Nomenclature functional dysregulation of the neuro-immune axis in preeclampsia

**Figure 1:**
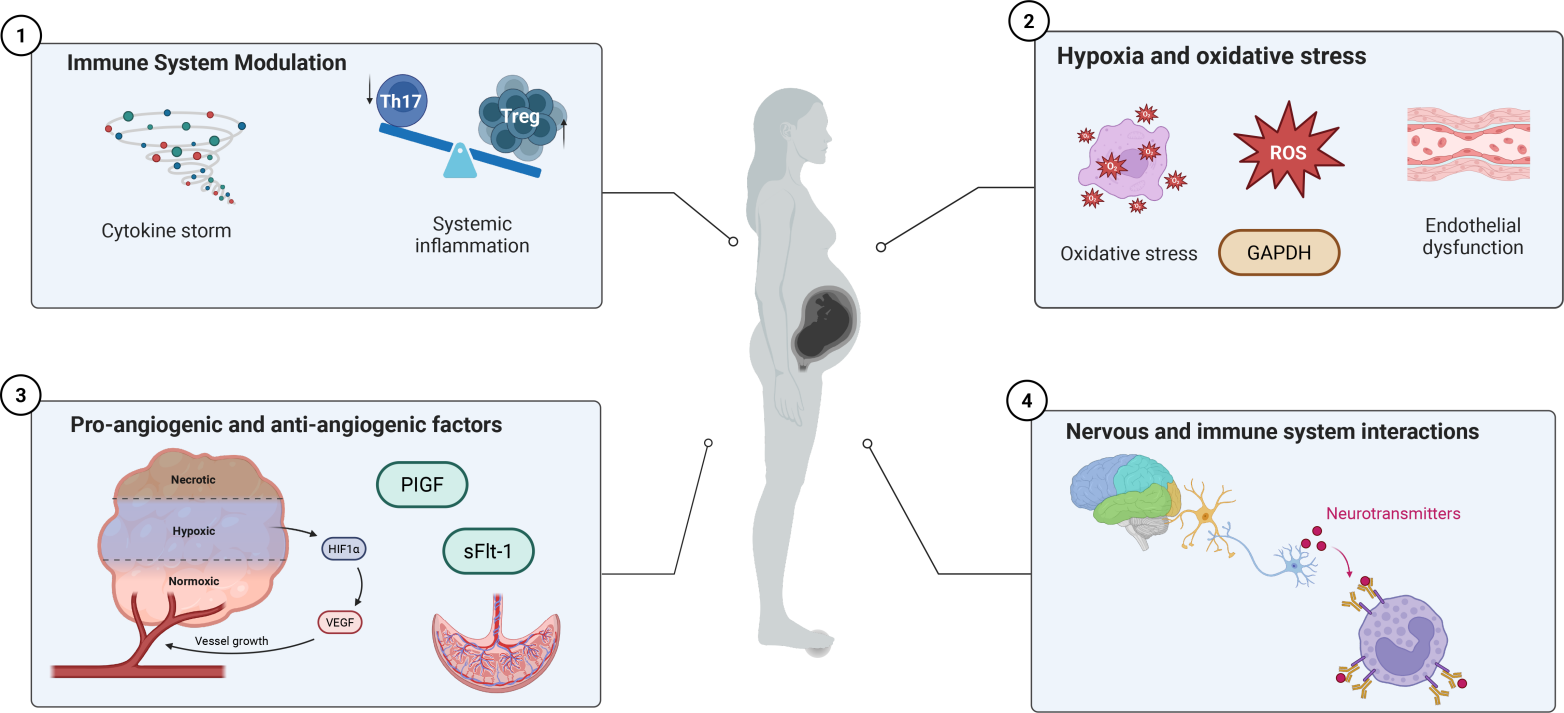
A Proposed Model of Systemic Neuro-Immune Crosstalk in Preeclampsia. An infographic featuring a central silhouette of a pregnant woman to illustrate a proposed model of systemic neuro-immune crosstalk in preeclampsia. Four interconnected panels detail: immune system modulation (cytokine storm and Th17/Treg balance), hypoxia and oxidative stress (ROS production), angiogenic imbalance (VEGF, sFlt−1, PlGF), and nervous-immune interactions (neurotransmitters). Arrows connect these components to demonstrate a self-amplifying loop leading to vascular pathology and endothelial dysfunction.

PE is characterized by a significant dysfunction in the neuro-immune axis (72). This dysregulation impacts all stages of the immune cell lifecycle, including development, distribution, and effector functions. Driven by disturbed neural signaling, this functional breakdown causes the systemic inflammation and endothelial damage (20). To visualize the systemic integration of these pathological pathways, we propose a comprehensive neuro-immune interaction model that drives the hallmark vascular dysfunction in preeclampsia (**Figure 1**).

### 4.1 Functional consequences of neuro-immune dysregulation

#### 4.1.1 Immune cell development and production

The development and maturation of immune cells, primarily in the bone marrow, are tightly regulated processes profoundly influenced by neuro-immune interactions (16). A key functional dysregulation in PE involves the aberrant influence of the sympathetic nervous system on hematopoiesis (85). Chronic stress and systemic inflammation activate sympathetic pathways, increasing neurotransmitter release like norepinephrine in the bone marrow microenvironment (86). This sympathetic overactivity directly stimulates hematopoietic stem and progenitor cells, driving myeloid hyperplasia—increased production of myeloid lineage cells (neutrophils, monocytes, macrophages) (58). In PE, circulating neutrophils and monocytes are often increased, activated, and pro-inflammatory (87). These cells significantly contribute to systemic inflammation and endothelial dysfunction. For instance, GATA1-mediated macrophage polarization via the TrkB/cGMP-PKG signaling pathway has been implicated in PE (88), suggesting a role for specific signaling cascades in immune cell development and function that could be influenced by neuro-immune interactions.

#### 4.1.2 Immune cell distribution and migration

**Figure 2:**
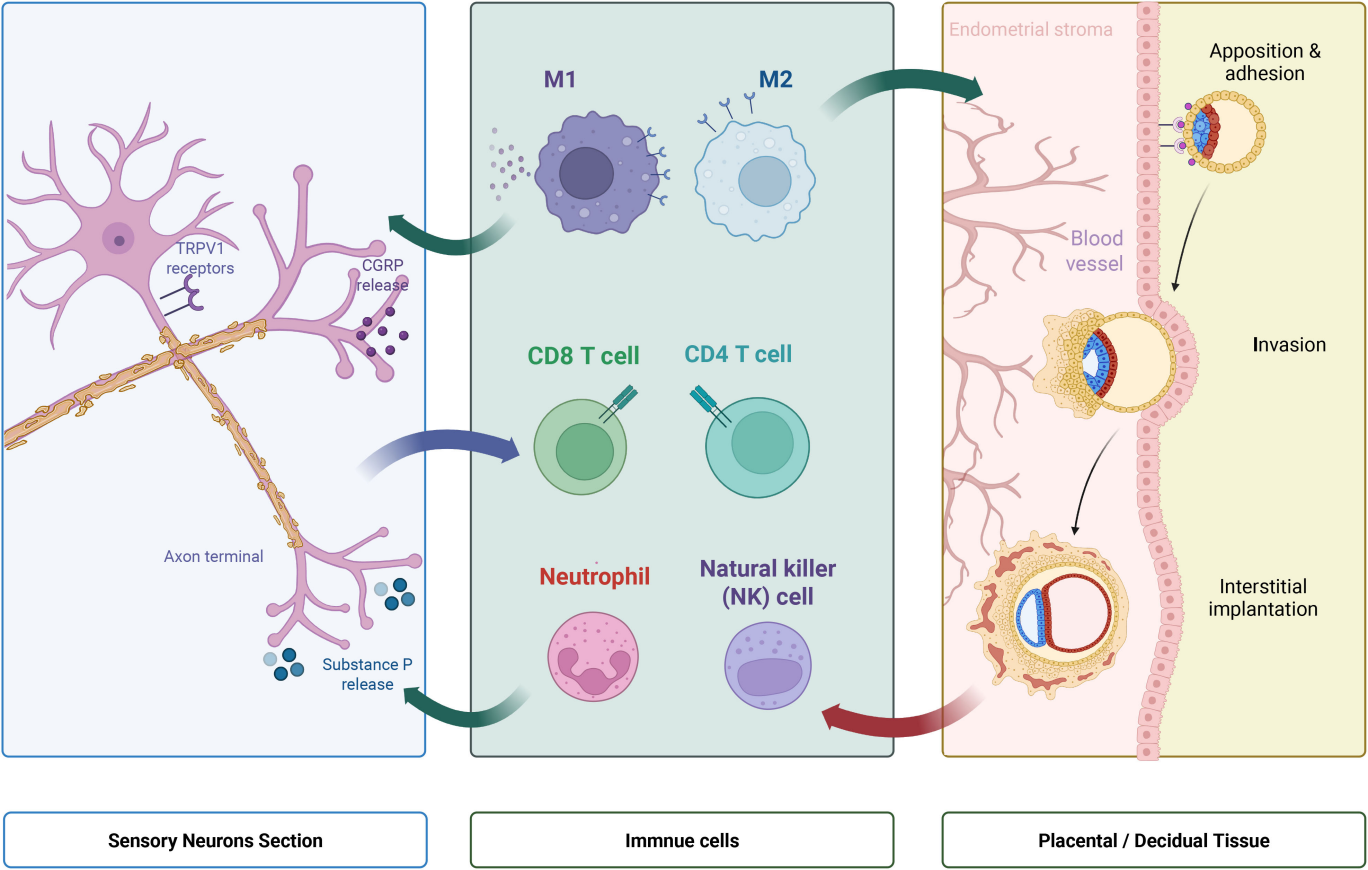
Sensory Neuro-Immune Regulation of the Maternal-Fetal Interface During Implantation. A schematic illustrating the sensory neuro-immune regulation of the maternal-fetal interface during implantation. Sensory neurons expressing TRPV1 receptors release neuropeptides like CGRP and substance P into the decidual microenvironment. These mediators interact with diverse immune cells, including NK cells, CD4^+^ and CD8^+^ T cells, neutrophils, and M1/M2 macrophages. The right portion displays stages of embryo apposition, adhesion, and interstitial invasion, highlighting how neuro-immune dialogue shapes early placentation.

Neurotransmitters (89) and neuropeptides (33) can directly influence the trafficking and homing of immune cells to specific tissues. In PE, altered neuro-immune signaling could lead to leukocyte redistribution, promoting the infiltration of pro-inflammatory immune cells into target organs such as the placenta, kidneys, and brain (48). The increased presence of activated immune cells, including macrophages and T cells, at the maternal-fetal interface and in the maternal circulation in PE suggests altered migratory patterns (90). This redistribution, guided by chemokines, cytokines, and neural signals, leads to inflammatory cell accumulation in specific organs. The altered neuro-immune environment also affects regulatory immune cell trafficking, such as Tregs, whose impaired function or reduced numbers correlate with PE (91). This imbalance, favoring pro-inflammatory cells, contributes to systemic inflammation and endothelial damage. The intricate crosstalk between sensory neurons and various immune cell subsets at the maternal-fetal interface during early placentation is illustrated in **Figure 2**.

### 4.2 Pathological neuro-immune signaling in preeclampsia

#### 4.2.1 Sensory and autonomic imbalance in PE

PE is characterized by a profound disturbance in sensory and autonomic neural regulation, though the causal direction remains a subject of intense investigation. While direct functional evidence of sensory neuron hyperexcitability (primarily derived from electrophysiological recordings in rodent models) suggests a state of neural sensitization (74), evidence in human PE remains largely inferential. In clinical cohorts, this state is inferred from indirect markers, such as the significant reduction in CGRP-positive sensory fiber density and the aberrant expression of ion channels (e.g., TRPV1, Nav1.8) in decidual tissues (37, 71). These anatomical and biochemical alterations correlate with increased sympathetic tone and impaired parasympathetic buffering, culminating in elevated vascular resistance (4). Distinguishing whether this neuro-immune imbalance is a primary etiological driver or a secondary response to the disease state is critical. Studies using Doppler flow and autonomic profiling have revealed that sympathetic overactivity correlates with early endothelial dysfunction and poor uteroplacental perfusion (92). These early shifts suggest an underlying neural susceptibility that may impair initial spiral artery remodeling. However, once the syndrome is established, secondary neural activity undoubtedly plays a major role. Placental hypoxia and the subsequent surge in pro-inflammatory cytokines act as potent stimuli that further sensitize sensory afferents and trigger systemic catecholamine release (90, 93). This creates pathological positive feedback, where the initial placental insult is amplified by a hyper-responsive nervous system. Therefore, PE is likely defined by an initial “neural stimulation” that is subsequently exacerbated by secondary hypoxia-driven inflammatory cascades, establishing a pro-hypertensive and pro-inflammatory environment.

#### 4.2.2 Altered neuropeptide and receptor signaling

Neuropeptides act as pivotal molecular mediators of neuro-immune crosstalk (33), and their imbalance is a recurring hallmark of PE. Circulating and placental levels of calcitonin gene-related peptide (CGRP) were found to be significantly lower in women with PE than in normotensive pregnancies, and this reduction was observed across gestational stages (94), with some studies reporting attenuated gestational increases in maternal serum CGRP in PE cohorts (95, 96). A research had also shown that CGRP receptor components, namely calcitonin receptor-like receptor (CRLR/CALCRL) and receptor activity-modifying protein 1 (RAMP1), were expressed in fetoplacental vessels and that CGRP-dependent vasorelaxation was significantly blunted in vessels from preeclamptic placentas (97).CGRP deficiency limits vasodilatory capacity, suppresses trophoblast migration, and impairs spiral artery remodeling, culminating in placental ischemia. Moreover, decreased expression of CGRP receptors (comprising CALCRL and RAMP1 (98)) has been detected in placental endothelium and decidual macrophages in PE (99). Conversely, Substance P and its receptor NK1R are upregulated, leading to enhanced mast cell degranulation, dendritic cell activation, and increased vascular permeability (100, 101). Contradictory findings across gestational stages and PE subtypes likely reflected the heterogeneous nature of the disease. Study of maternal CGRP concentrations demonstrated that although CGRP increased with advancing gestation in both normotensive and PE pregnancies, the degree of increase was significantly less in PE, and postpartum levels declined in both groups (94). Taken together with evidence that fetoplacental CGRP responsiveness was compromised in late pregnancy, these data suggested that neuropeptide imbalance in PE was dynamic and stage-specific, with more pronounced deficits in early-onset or severe PE relative to late-onset or mild forms.

Vasoactive intestinal peptide (VIP), another critical neuropeptide promoting Th2 and Treg balance (102), is likewise diminished in PE, compromising the anti-inflammatory microenvironment necessary for fetal tolerance (102). The imbalance between vasodilatory (CGRP, VIP) and pro-inflammatory (Substance P, neurokinin A) signaling produces a neuropeptide milieu favoring vasoconstriction and immune activation (103). This shift not only disrupts vascular homeostasis but also reshapes immune phenotypes toward Th1/Th17 polarization and innate cell activation (102), further amplifying endothelial injury and hypertension. Loss of CGRP and VIP signaling reduces the angiogenic function of NK cells, while heightened sympathetic input and Substance P enhance their cytotoxicity via NK1R-dependent pathways (104). Simultaneously, decidual macrophages polarize toward an M1-like inflammatory state, releasing TNF-α, IL-1β, and reactive oxygen species that further impair endothelial integrity and trophoblast invasion (105, 106).

Neutrophils also show excessive activation in PE, forming neutrophil extracellular traps (NETs) that occlude micro vessels and propagate oxidative stress (107, 108). Normally, CGRP and VIP suppress neutrophil adhesion and reactive oxygen species (ROS) production (99, 102), but in PE this restraint is lost, possibly due to receptor downregulation and oxidative damage to peptidergic terminals (7). Together, these processes establish a self-reinforcing loop: neuronal dysfunction triggers innate immune activation, which in turn releases cytokines and ROS that further damage neurons (109), propagating neuro-immune injury at the uteroplacental interface (110, 111).

## 5 Advanced methodologies and emerging concepts in neuro-immune preeclampsia research

**Figure 3:**
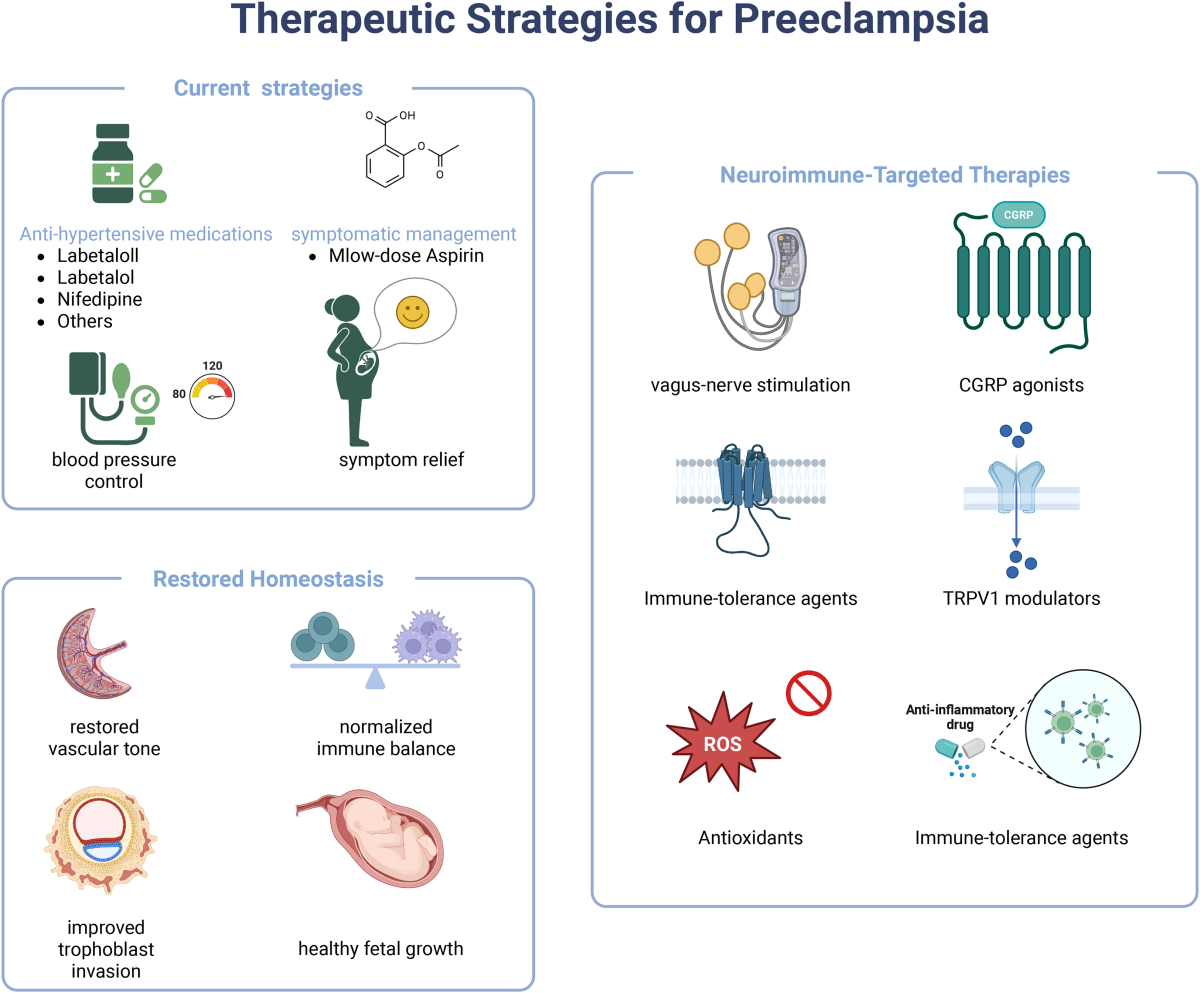
The Therapeutic Landscape of Preeclampsia: From Symptomatic Control to Neuro-Immune Modulation. An infographic mapping the therapeutic landscape of preeclampsia, transitioning from symptomatic control to neuro-immune modulation. Current strategies focus on symptomatic management using antihypertensives and low-dose aspirin. The goal of restoring homeostasis includes normalizing vascular tone, rebalancing immunity, and improving placental function and fetal growth. Emerging neuro-immune-targeted therapies include bioelectronic interventions like vagus nerve stimulation and pharmacological agents such as CGRP agonists and TRPV1 modulators to restore physiological crosstalk and address the underlying neuro-immune pathophysiology.

The complexity of the neuro-immune axis in PE necessitates advanced methodologies and the exploration of novel concepts to unravel its intricate mechanisms. Rapid advancements in technological innovations are revolutionizing PE research, providing unprecedented opportunities to dissect the neuro-immune axis at molecular and cellular resolution. Hence, we outline the therapeutic evolution from traditional symptomatic control toward mechanism-driven, neuro-immune-targeted interventions (**Figure 3**). The core contribution advantages and disadvantages (or challenges) of emerging therapeutic technologies designed to modulate the neuro-immune axis in preeclampsia are consolidated in **Table 2**. 

**Table 2:** Therapeutic technologies targeting the neuro-immune axis in preeclampsia.

Technology	Core contribution to PE neuro-immune axis research	Advantages	Disadvantages/challenges
Single-Cell & Spatial Multiomics	Unravels cellular heterogeneity, cell-cell communication, and gene expression changes at maternal-fetal interface and other organs, identifying specific dysregulated immune and trophoblast subsets.	High resolution, identifies novel cell types/states, maps spatial interactions.	High cost, complex data analysis, limited sample availability.
Organoid Models	Provides *in vitro* platforms for studying trophoblast function, maternal-fetal interactions, and testing drug effects in a physiologically relevant context; potential for neural organoids with integrated immune cells.	Mimics *in vivo* physiology, enables mechanistic studies, drug screening.	May not fully replicate complex *in vivo* environment, ethical considerations for human tissue.
Bioelectronic Medicine	Modulates neural circuits (e.g., vagus nerve) to control immune responses and inflammation, offering precise, targeted therapeutic avenues.	Non-pharmacological, precise control, potential for personalized therapy.	Safety in pregnancy, optimal stimulation parameters, long-term effects.
AI & Machine Learning	Integrates multi-modal data (omics, clinical, imaging) to identify biomarkers, predict disease risk, and uncover complex patterns in neuro-immune dysregulation.	High predictive accuracy, identifies subtle patterns, accelerates biomarker discovery.	Data quality/bias, interpretability of models, requires large datasets.
Exosomal & MicroRNA	Identifies non-coding RNAs (miRNAs, piRNAs) as potential biomarkers and regulators of gene expression in neuro-immune pathways, affecting trophoblast function and inflammation.	Non-invasive biomarkers, insights into regulatory mechanisms, therapeutic targets.	Challenges in standardization, specificity, and validation in large cohorts.

### 5.1 Omics approaches and single-cell technologies

The advent of omics technologies, particularly scRNA-seq, has revolutionized the study of complex biological systems like the maternal-fetal interface and systemic immune responses in PE (112). Traditional bulk RNA sequencing often masks the heterogeneity of cell populations, making it difficult to identify cell-type-specific changes. However, scRNA-seq allows for the characterization of gene expression profiles at the individual cell level, providing a granular view of cellular states and interactions (113).In PE research, scRNA-seq has been instrumental in deciphering the immune disturbances landscape and identifying specific cell clusters with dysregulated gene expression (30, 114), highlighting the power of scRNA-seq in identifying specific cellular culprits and molecular pathways.

Beyond transcriptomics, spatial multiomics approaches, combining spatial transcriptomics (ST) (115) and spatial metabolomics (SM) (116), are providing unprecedented insights into the spatial organization of cells and their metabolic activities within tissues. A multiomics single-cell atlas of the human maternal-fetal interface has revealed the full trajectory of trophoblast differentiation and predicted cell-cell communication events in trophoblast invasion (17). Similarly, spatial metabolomics and transcriptomics have revealed cell-type-specific metabolic reprogramming in the placenta, particularly in glycerophospholipid and sphingolipid metabolism, influencing trophoblast differentiation (116). These technologies are crucial for understanding localized neuro-immune interactions, as they can map the expression of neurotransmitter receptors, neuropeptides, and immune mediators within specific cellular niches.

### 5.2 Organoid and animal models for neuro-immune studies

To complement human tissue analysis, advanced *in vitro* and *in vivo* models are essential for mechanistic studies of the neuro-immune axis in PE. Trophoblast organoids, offer a genetically stable, long-term culture system that allow for controlled experimentation on trophoblast interactions with the maternal environment and immune cells, providing a platform to investigate how neuro-immune signals might influence placental development and dysfunction in PE (117). The advantage of organoids is their human origin and ability to replicate complex tissue architecture, reducing reliance on animal models for certain aspects of human biology (118).

Animal models, particularly mouse models of PE, are indispensable for studying systemic neuro-immune interactions and testing therapeutic interventions. Various mouse models exist that mimic aspects of PE, such as hypertension, proteinuria, and placental dysfunction (119). For example, studies using models of gut dysbiosis or specific microbial components show they can induce PE-like symptoms. Importantly, these symptoms can be improved by treatments that target the gut microbiota (120). To date, no optogenetic or chemogenetic interventions have been reported in animal models of preeclampsia; however, substantial evidence from inflammatory and neuro-immune research indicates that precise manipulation of defined neural circuits can regulate immune and inflammatory responses. In a mouse inflammatory pain model, optogenetic activation of neurons in the somatosensory cortex affected pain responses and inflammatory signaling pathways, showing that optogenetic modulation of electroacupuncture analgesia in a mouse inflammatory pain model (121). In rat model, rat with chemogenetic excitation or inhibition of the medial prefrontal cortex (mPFC) underwent either a brief ineffective stress or a prolonged effective stress. Specifically, chemogenetic enhancement of neuronal activity in the mPFC negated the impairing effects of a 60-min stress on object recognition memory (122). Hence, the development of more sophisticated animal models that integrate neural components with placental and systemic immune dysfunction is crucial for advancing this field.

### 5.3 Bioelectronic medicine and neuro-immune modulation tools

The growing understanding of neuro-immune interactions is driving the field of bioelectronic medicine (123), which uses electrical or optical stimulation to modulate neural circuits and control immune responses (124). Tools like vagus nerve stimulation (VNS) are being explored for their anti-inflammatory potential, and noninvasive ultrasound stimulation has shown efficacy in modulating splenic neuro-immune regulation to treat inflammation (24, 125, 126). These technologies offer precise ways to manipulate neural pathways that influence immune function, providing novel therapeutic avenues for PE.

### 5.4 Artificial intelligence and machine learning

AI (artificial intelligence) (127, 128) and machine learning algorithms (129, 130) are increasingly being applied to large datasets, including omics data and clinical records, to identify novel biomarkers, predict disease risk, and uncover complex patterns in PE. For example, AI models using ECG data have demonstrated high accuracy in detecting and predicting PE, even weeks before diagnosis (127). Machine learning-based methods using scRNA-seq have identified pathological cell subpopulations and predicted PE risk with high accuracy, also discovering driver genes that may mediate inflammation (129). Deep learning algorithms applied to retinal fundus images also show promising predictive value for early PE detection (131). These computational approaches are crucial for integrating multi-modal data and translating complex biological insights into clinically actionable tools.

### 5.5 Exosome and microRNA research

Exosomal small RNA profiling in first-trimester maternal blood is exploring early molecular pathways of preterm PE, identifying differentially expressed RNAi-related species are microRNAs (miRNAs) and Piwi-associated small RNAs (piRNAs) as potential biomarkers and linking them to immune pathways (132). For example, miR-214-3p deletion has been shown to alleviate PE-like symptoms by regulating PlGF and eNOS (133), while miR-223-3p downregulates the inflammatory response in PE placenta via targeting NLRP3 (134). Exosomal MicroRNAs also show promise as non-invasive biomarkers for diagnosis, prediction, and treatment, regulating mesenchymal stem cells, trophoblasts, and nitric oxide production (135). These studies highlight the potential of small RNAs as both biomarkers and therapeutic targets.

These technologies collectively accelerate the understanding of the neuro-immune axis in PE, moving towards precision medicine where diagnostics and therapies can be tailored to individual patients’ neuro-immune and metabolic profiles.

## 6 Limitations and future perspectives

PE, once primarily viewed as a vascular disorder, is now unequivocally understood as a complex systemic disease driven by intricate neuro-immune-vascular dysregulation (72). This review has meticulously dissected the neuro-immune axis in PE, highlighting its physiological basis in normal pregnancy and the profound spatial and functional aberrancies that characterize the disease. We established that the maternal-fetal interface serves as the primary hub where aberrant neuro-immune crosstalk (136), often exacerbated by sympathetic overactivity, initiates the pathological cascade of impaired placentation and systemic inflammation (43). This local dysfunction remotely impacts peripheral immune organs (like the bone marrow and spleen) (64, 86), which, under altered neural signaling, contribute to dysregulated immune cell development, distribution, and effector functions. Ultimately, the CNS becomes both an amplifier and an effector, manifesting in a spectrum of neurological symptoms (23, 66), including eclamptic seizures. Functionally, PE is marked by sympathetic nerve signals driving myeloid hyperplasia, leading to altered leukocyte redistribution and target organ infiltration (53), culminating in a detrimental cytokine storm and endothelial vessel attack.

The intricate crosstalk between neuro-immunity and metabolism further complicates the disease, highlighting the need for integrated approaches. Technological innovations, including single-cell and spatial multi-omics (114), organoid models (117), bioelectronic medicine (123), and AI (127), are rapidly advancing our understanding, enabling high-resolution mechanistic insights and novel biomarker discovery. Clinically, targeting the neuro-immune axis offers promising avenues for developing comprehensive biomarker panels, pioneering neuroregulatory therapies like vagus nerve stimulation, and repurposing existing pharmacological agents (such as dexmedetomidine (137), beta-blockers (138), and JAK inhibitors (139)).

Despite significant progress, several critical research gaps remain that warrant focused future investigation: Firstly, the exact neural circuits, specific neurotransmitters, and their downstream molecular targets driving immune dysregulation at the maternal-fetal interface and in peripheral organs in PE are not fully elucidated. Although no published optogenetic or chemogenetic intervention has yet been applied in PE animal models, numerous studies in inflammatory and neuro-immune systems demonstrate that targeted modulation of defined neural circuits can alter immune or inflammatory outcomes. Future research needs to map these neuro-immune circuits with greater precision, potentially using advanced neuroimaging, optogenetics, and chemogenetics in relevant animal models. Secondly, studies should integrate multi-omics data from pregnancy through the postpartum period and beyond to identify persistent neuro-immune signatures and their correlation with later-life diseases. Furthermore, recent evidence underscores the pivotal role of fetal sex as a biological variable in PE pathogenesis. A nested case-control indicates that fetal sex is associated with differences in maternal cytokines in preeclamptic vs control pregnancies (140). Another research also demonstrates that male and female placentas show different molecular and metabolic responses in PE, supporting sexual dimorphism in placental lipid metabolism (141). Future neuro–immune studies in preeclampsia should incorporate NIH rigor guidelines, including randomization, blinding, and *a priori* power calculations, as well as explicit consideration of fetal sex as a biological variable. Thirdly, the complex interplay between neuro-immunity and metabolism in PE needs further unraveling. Future studies should investigate how metabolic dysregulation (such as, gestational diabetes, obesity) directly influences neuro-immune pathways and, conversely, how neuro-immune dysregulation contributes to metabolic disturbances. This could involve integrated multi-omics analyses of gut microbiota, host metabolism, and neuro-immune markers, leading to novel therapeutic targets addressing both metabolic and inflammatory aspects. Lastly, PE is a heterogeneous syndrome. Future research must move towards personalized medicine, utilizing advanced computational tools like AI and machine learning to integrate diverse patient data (clinical, genetic, omics, imaging) to identify specific neuro-immune endotypes. Notably, promising preclinical findings regarding neuroregulatory therapies and repurposed drugs need rigorous testing in well-designed clinical trials. These trials must prioritize safety for both mother and fetus, establish optimal dosing and timing, and evaluate efficacy in diverse patient populations. This will enable tailored diagnostic and therapeutic strategies optimized for individual patients.

## 7 Conclusion

PE is a multi-system disorder in which placental dysfunction, immune activation, and endothelial injury remain core mechanisms. However, acute neurological phenotypes (e.g., eclampsia and cerebral autoregulatory failure) were not fully explained by placental or vascular mechanisms alone. Hence, the neuro-immune axis represents a pivotal dimension of PE pathophysiology. Dysfunction of cerebral autoregulation has been proposed as a key pathophysiological process underlying these neurological manifestations in PE and eclampsia (81). Evidence of altered autonomic control in PE further implicates central neural pathways in systemic cardiovascular responses, supporting a role for neural regulation in specific disease expressions (79).

Based on this framework, we propose several conditional and falsifiable predictions for future investigation: First, targeted modulation of central autonomic or sensory integration circuits may reduce neurological manifestations, such as eclampsia or failure of cerebral autoregulation, in preclinical models even when placental pathology persists. Second, subtypes of PE that are characterized by acute neurovascular instability are expected to show stronger signatures of autonomic imbalance than subtypes that are driven primarily by placental angiogenic dysfunction, as measured using standardized autonomic biomarkers. Third, individuals with heightened baseline neuroimmune or autonomic sensitivity are predicted to exhibit greater neurological vulnerability in response to similar placental stressors compared with individuals who have more robust autonomic regulation.

To ensure healthier pregnancies and lives afterward, PE research must embrace its systemic neuro-immune nature and translate complex scientific knowledge into practical treatment measures.
